# Emotional freedom techniques for treating post traumatic stress disorder: an updated systematic review and meta-analysis

**DOI:** 10.3389/fpsyg.2023.1195286

**Published:** 2023-08-10

**Authors:** Peta Stapleton, Kevin Kip, Dawson Church, Loren Toussaint, Jacqui Footman, Pat Ballantyne, Tom O’Keefe

**Affiliations:** ^1^School of Psychology, Bond University, Gold Coast, QLD, Australia; ^2^Health Services Division, University of Pittsburgh Medical Center, Pittsburgh, PA, United States; ^3^National Institute for Integrative Healthcare, Fulton, CA, United States; ^4^Department of Psychology, Luther College, Decorah, IA, United States; ^5^EFT International, Tiverton, Devon, United Kingdom

**Keywords:** emotional freedom techniques, PTSD, trauma, empirically supported treatment, evidence-based

## Abstract

**Introduction:**

Clinical Emotional Freedom Techniques (EFT) is a psychophysiological intervention that includes cognitive and somatic elements, utilizing techniques from both Cognitive Behavioral Therapy (CBT) and Prolonged Exposure therapy (PE). Because only a single meta-analysis existed examining EFT for PTSD, this systematic review and meta-analysis represents an update.

**Method:**

Ten databases were searched for quantitative reviews and randomised clinical trials, and six met inclusion criteria.

**Results:**

Study quality and effect size were evaluated and the results demonstrated that treatment with Clinical EFT, when compared to wait list, usual care, or no treatment controls, resulted in significant and large effect sizes, ranging from 1.38 to 2.51. When compared to active controls, effect sizes ranged from −0.15 to 0.79, producing treatment results similar to other evidence-based therapies.

**Discussion:**

Limitations are presented and considerations for further research are proposed.

## Public health significance

Post-traumatic stress disorder (PTSD) is an accelerating mental health challenge worldwide (von der Warth et al., 2020). An estimated 30 % of those returning from a combat zone will develop PTSD (Shalev et al., 2017), and 6.8 % of Americans are likely to suffer from PTSD in their lifetime (Harvard Medical School, 2007), with sexual and other interpersonal violence accounting for nearly two-thirds of cases (Julia, 2022). Half of these individuals will never engage in treatment or intervention (Kessler et al., 2017). PTSD may develop after an injury or assault, a shooting incident, a natural disaster, the sudden death of a loved one, or witnessing the death or serious injury of another (Julia, 2022). The often-debilitating symptoms of PTSD include flashbacks, nightmares, intrusive thoughts, severe anxiety, hypervigilance, sleep disturbance, physical aggression, and poor concentration (Bisson et al., 2015). These symptoms are associated with deterioration in the personal, social, and financial domains of life.

Both psychological and pharmacological interventions are available to treat PTSD. Cognitive and exposure therapies have been demonstrably effective, with a review of early studies concluding that: “The current literature reveals robust evidence that CBT is a safe and effective intervention for both acute and chronic PTSD following a range of traumatic experiences in adults, children, and adolescents” (Kar, 2011, p. 167). The authors caution, however, that “nonresponse to CBT by PTSD can be as high as 50%, contributed to by various factors, including comorbidity and the nature of the study population” (p. 167). A review of 36 randomized controlled trials (RCTs) found that two-thirds of military personnel or veterans treated for PTSD still met the diagnostic criteria for PTSD following cognitive processing and prolonged exposure treatments (Steenkamp et al., 2015). While pharmacological treatments (e.g., selective serotonin reuptake inhibitors) do reduce symptoms, relapse is common upon cessation of treatment (Alexander, 2012). Clinical guidelines typically recommend that first-line treatments should be trauma-focussed psychotherapies (e.g., American Psychological Association, 2017).

## Background

### Body-based interventions for PTSD

While research supports the use of talk-oriented therapies in the treatment of trauma, the clinical advantages of bringing somatic elements into rehabilitation are increasingly being recognized. Van der Kolk originally proposed that the changes in biological responses noted after trauma indicated that trauma may be stored as a bodily memory, suggesting that treatments should utilize somatic interventions (van der Kolk, 1994). Somatic approaches can target not only the muscles and fascia, but also the neurological correlates of trauma. Ogden et al. (2006), propose a “bottom-up,” body-centred approach to trauma.

The focus of this analysis is Clinical Emotional Freedom Techniques (Clinical EFT), a therapeutic modality that incorporates both cognitive and somatic elements. In the late 1990s, the American Psychological Association (APA) Division 12 Task Force for Empirically Validated Treatments published a set of seven standards (based on Chambless and Hope, 1996 and Chambless and Hollon, 1998). These were designed as a guide for evaluating the quality of evidence supporting the efficacy of a given therapeutic modality. They placed particular emphasis on randomized controlled trials (RCT), with two high-quality independent RCTs being required to establish a therapy as “efficacious.”

For two decades, the Chambless and Hope (1996) guidelines provided a stable, well-defined, published set of common standards by which the efficacy of a therapeutic technique could be judged. A 2013 systematic review compared extant research in Clinical EFT against the standards and found that the method met the criteria as an “evidence-based” practice for anxiety, depression, phobias, and PTSD (Church, 2013a). Following publication of the Chambless and Hope (1996) criteria, most randomized controlled trials of Clinical EFT were explicitly designed to meet them. A recent systematic review identified 56 randomized controlled trials of Clinical EFT (Church et al., 2022), many citing and crediting the Chambless and Hope (1996) criteria for their design in the Methods section. Many therapeutic modalities other than Clinical EFT also performed RCTs meeting the criteria. These standards thus influenced the design of hundreds of studies and contributed to an entire generation of high-quality research.

### Emotional freedom techniques

EFT was developed in the 1980s, and the standardized treatment manual for Clinical EFT was introduced in the early 1990s. EFT is a psychophysiological intervention that includes cognitive and somatic elements. It utilizes techniques from both Cognitive Behavioral Therapy (CBT) and Prolonged Exposure therapy (PE). These include awareness building, imaginal exposure, cognitive reframing, preframing, and systematic desensitization. To this it adds the somatic ingredient of acupressure. Rather than using acupuncture needles, practitioners stimulate, or teach their clients to self-stimulate, acupuncture points (acupoints) by tapping on them with their fingertips, a practice drawn from Eastern healing traditions such as acupressure, Qigong, and Shiatsu. For this reason, EFT is popularly referred to simply as “tapping.”

Peer-reviewed papers exploring psychotherapies that utilize acupoint tapping include five meta-analyses of acupoint tapping protocols, eight meta-analyses of multiple approaches that include a tapping treatment, 15 other systematic reviews, 69 RCTs, 56 clinical trials using standardized outcome measures but no control group, 24 case studies, 26 reports describing systematic observations, 17 mixed-method clinical trials that included a tapping component, and 88 articles addressing clinical procedures, theory, mechanisms, or related issues (Feinstein, 2022a). More than 90 additional clinical trials investigating EFT or close variations have been published in non-English language journals (Freedom et al., 2022). The APA has been providing continuing education credits for courses in EFT since 2011, and the AMA (American Medical Association) and ANCC (American Nurses Credentialing Commission) since 2013.

The Clinical EFT protocol begins with obtaining from the client a Subjective Units of Distress (SUD) score on the target issue (after Wolpe, 1969). SUD scores can range from zero (indicating no distress or neutral) to 10 (indicating the highest possible level of distress). The client then uses a “Setup Statement” that involves naming the distressing event while pairing the memory with a statement of self-acceptance (Church and Feinstein, 2017). This combines exposure with cognitive framing. While the setup statement is spoken, an acupoint on the side of the hand is tapped. Eight other acupoints are then tapped while repeating an emotive “Reminder Phrase” designed to evoke maximum affect. Wording is adjusted for subsequent rounds of tapping based on shifts in the SUD score.

### Physiological shifts following clinical EFT sessions

Early research showed clinically beneficial electroencephalogram (EEG) changes following EFT treatments motor vehicle accident victims suffering from PTSD had increased 13–15 Hz amplitude over the sensory motor cortex, decreased right frontal cortex arousal and an increased 3–7 Hz/16–25 Hz ratio in the occiput (Swingle et al., 2010). Similar outcomes have been observed for traumatic brain injury (Craig et al., 2009), claustrophobia (Lambrou et al., 2002), and seizure disorders (Swingle, 2005). Clinical EFT has also been associated with the regulation of blood pressure, heart rate, and immune markers (Bach et al., 2019). A study by Church et al. (2012) showed greater reductions in cortisol levels after a single tapping session than after a session of talk therapy, a finding replicated by Stapleton et al. (2020).

More sophisticated designs have measured changes in gene expression (Church et al., 2016; Maharaj, 2016) as well as epigenetic signaling molecules named microRNAs (Yount et al., 2019). Yount et al. (2019) identified three microRNAs with expression levels that correlated significantly with psychological tests of PTSD. Neural changes using functional magnetic resonance imaging (fMRI) have identified a reduction in activity in the reward areas of the brain (Stapleton et al., 2019) that were associated with corresponding reductions in food cravings, emotional eating and increased restraint ability (Stapleton et al., 2019).

Decreased connectivity between the medial prefrontal cortex (a pain modulating area) and bilateral grey matter areas in the posterior cingulate cortex and thalamus (Stapleton et al., 2022) was evident after a 6-week EFT intervention for chronic pain sufferers and corresponded with participants’ self-reported decreases in severity and intensity of pain, psychological distress and improvements in happiness and satisfaction in life (Stapleton et al., 2022). Acupoint tapping increased amygdala activation and decreased hippocampus activation in flight-phobic participants (Wittfoth et al., 2022), while similar amygdala activation and decreased ventral anterior cingulate cortex activation was identified during emotion regulation tasks (Wittfoth et al., 2020). These effects were contrary to the previous EFT studies that resulted in down-regulation of neural activity and areas, and may seem counterintuitive, however symptom severity and negative affect were still reduced in participants and the authors proposed the split of attention between the emotional stimuli and physiological stimulation of acupoints, allows one to process the negative stimulus for new integration. This process does not default to distressing responses, but instead allows for higher limbic activation (in the amygdala) and decreased prefrontal activation.

Whether tapping makes a clinical contribution, or whether EFTs observed effects are due to its cognitive and exposure components, has been investigated in six studies and a meta-analysis (Church et al., 2018a). The six individual studies all found that tapping on acupoints was more effective than tapping on sham points or other active controls. The meta-analysis identified a large treatment effect for the full Clinical EFT protocol, and a moderate effect superior to controls. The results of these investigations show that tapping is an active rather than an inert ingredient in the procedure.

### The contribution of acupuncture and acupressure

Because the stimulation of acupuncture points is the primary somatic ingredient of EFT, a brief overview of the evidence base for acupuncture and acupressure (sometimes referred to as “acupuncture without needles”), is warranted. Over 13,000 studies and more than 2,500 reviews of acupuncture and acupressure appear in the literature (Ma et al., 2016). In 2017 the Acupuncture Evidence Project (McDonald and Janz, 2017) drew on and expanded two prior comprehensive literature reviews: one conducted for the United States Department of Veterans Affairs in 2013, the other for the Australian Department of Veterans’ Affairs in 2010. The Acupuncture Evidence Project evaluated existing studies using the National Health and Medical Research Council (NHMRC) levels of evidence criteria and the Cochrane GRADE system for assessing risk of study bias. The aim was to present the current state of evidence and how the quality and quantity of research had changed from 2005 to 2016. Of the 122 medical and psychiatric conditions reviewed across 14 broad clinical areas, research supported the effectiveness of acupuncture for 117 of them, with the evidence for 46 of them being at “moderate or high quality.” Only five of the 122 conditions were rated at “no evidence of effect.” An important trend identified was that in the 11-year period covered by the review, the level of evidence had increased for 24 conditions, corroborating earlier findings.

A core concept of acupuncture is that stimulating electrically sensitive points on the skin sends impulses to related organs along “energy pathways” known as *meridians.* An obstacle to the acceptance of acupuncture within the medical community has been the failure to find evidence of the meridians in the nervous system, musculoskeletal system, circulatory system, or other known anatomical structures (Leskowitz, 2018). Research in the past two decades, however, has lent support to the hypothesis that the meridian system is embedded in the body’s interstitial connective tissue. For instance, Langevin and Yandow (2002), using ultrasound imagery, found that 80 percent of the acupuncture points and 50 percent of the meridian intersections, as identified in traditional acupuncture theory, coincide with connective tissue planes in the arm. Earlier studies also suggested anatomical correspondences with the meridians, such as when injections of radioactive tracer dye into acupuncture points traveled along pathways that coincided with traditional descriptions of the meridians (de Vernejoul et al., 1992; Darras et al., 1993).

Individual acupoints have been shown to have less electrical resistance, and thus greater electrical conductivity, than adjacent points (Li et al., 2012). Through a well-established process known as mechanosensory transduction, cells can convert a mechanical stimulus (in the case of acupuncture and acupressure, needling or tapping) into electrical activity (Gillespie and Walker, 2001). The electrical signals generated by tapping are presumably transmitted along the meridian pathways that have been found to be contained within the body’s interstitial tissue, which has a high concentration of collagen. Because collagen is a semi-conductor, the rapid transmission of electrical impulses along the body’s connective tissue provides a plausible anatomical explanation for the action of acupoint stimulation. A systematic review identified 66 studies that compared acupressure on actual points with the stimulation of sham points (Tan et al., 2015). Pressure on actual points was found to be more effective for many health issues than sham treatment. This brief overview of some of the known mechanisms involved in acupoint tapping is presented to suggest that the somatic element of Clinical EFT, acupressure, rests on an established anatomical base.

### Clinical EFT for PTSD

An extensive body of research examining Clinical EFT for PTSD has accumulated. The populations represented in the research include war veterans, victims of sexual violence, the spouses of PTSD sufferers, motor accident survivors, prisoners, hospital patients, adolescents, and survivors of natural and human-caused disasters (Sebastian and Nelms, 2017; Feinstein, 2022b). Consistent clinical outcomes imply that the intervention is generalisable to a variety of settings and populations. The single meta-analysis for Clinical EFT and PTSD indicated a large pre- to post-treatment effect (*d* = 2.96) in four to 10 sessions (Sebastian and Nelms, 2017). Meta-analyses for depression (Nelms and Castel, 2016) and anxiety (Clond, 2016) have also yielded large pre- to post-treatment effect sizes (*d* = 1.31, *d* = 1.23 respectively).

In the seven studies reported in the existing meta-analysis of EFT for PTSD (Sebastian and Nelms, 2017), the mean dropout rate (defined as those who withdrew from the study or were lost to follow-up) was less than 10% of participants. EFT has also been included in conventional intervention programs, such as the “Warrior Combat Stress Reset Program” at Fort Hood, the largest US military base, using EFT and EMDR for the remediation of PTSD (Libretto et al., 2015). This particular program reported dropouts of less than 10 soldiers out of 1,400 over the life of the program. The 2019 Practice Guidelines for Clinical Treatment of Complex Trauma in Australia updated its recommendation to include EFT (Kezelman and Stavropoulos, 2019) noting that the method is nationally and internationally endorsed. The National Institute for Health and Care Excellence (NICE) in the United Kingdom has created a new category for treating PTSD, termed “Combined Somatic and Cognitive Therapies,” which is comprised of EFT and Thought Field Therapy (the precursor to EFT). NICE’s evaluation identified preliminary evidence for this category and indicated it is now one of its four research priorities.^1^

Given the increase in research and time elapsed since the initial meta-analysis of EFT for PTSD, it was timely to consider an update.

### Methods and procedures

Seven volunteers were recruited as reviewers, including one student member. Step 1 of the evaluation included an examination of the single existing meta-analysis of EFT in treating PTSD, as well as systematic reviews and randomized clinical trials. These were all evaluated for quality and effect sizes, presented in Step 2. Our final recommendation was determined after conducting this systematic review of published studies. The methods described were conducted according to the checklist of the Preferred Reporting Items for Systematic Reviews and Meta-Analyses (PRISMA; Moher et al., 2015) and the reporting standards of APA’s Publications and Communications Board Task Force Report (Levitt et al., 2018). The review methods were established with the committee members prior to the conduct of the review (November 2021) and there were no significant deviations.

### Search strategy and sources

Because only a single meta-analysis existed examining EFT for PTSD, this evaluation included systematic reviews, randomized controlled trials, and quantitative reviews. Unpublished literature was also included in order to represent the most current research.

We searched 10 databases including CINAHL, PsychInfo, Science Direct, Web of Science, Core Group, Embase, PubMed, Trip, Medline, and the Cochrane Database. Four broad search terms were used in the initial round with each database, including:

1. emotional freedom technique.mp. [mp = title, abstract, heading word, table of contents, key concepts, original title, tests and measures, mesh word] OR.
2. tapping.mp. [mp = title, abstract, heading word, table of contents, key concepts, original title, tests and measures, mesh word] OR.
3. acupoint.mp. [mp = title, abstract, heading word, table of contents, key concepts, original title, tests and measures, mesh word] OR.
4. meridian.mp. [mp = title, abstract, heading word, table of contents, key concepts, original title, tests and measures, mesh word].

Within each match, five secondary terms were searched, including:

1. PTSD.mp. [mp = title, abstract, heading word, table of contents, key concepts, original title, tests and measures, mesh word] OR.
2. trauma.mp. [mp = title, abstract, heading word, table of contents, key concepts, original title, tests and measures, mesh word] OR.
3. posttraumatic stress disorder.mp. [mp = title, abstract, heading word, table of contents, key concepts, original title, tests and measures, mesh word] OR.
4. distress.mp. [mp = title, abstract, heading word, table of contents, key concepts, original title, tests and measures, mesh word] OR.
5. stress.mp. [mp = title, abstract, heading word, table of contents, key concepts, original title, tests and measures, mesh word].

Within each of these matches, three additional terms were searched, including:

1. meta analysis. [mp = title, abstract, heading word, table of contents, key concepts, original title, tests and measures, mesh word].
2. systematic review. [mp = title, abstract, heading word, table of contents, key concepts, original title, tests and measures, mesh word].
3. clinical trial (randomized controlled or clinical trial). [mp = title, abstract, heading word, table of contents, key concepts, original title, tests and measures, mesh word].

The inclusion criteria were: each study must be an RCT investigating the use of EFT for treating the symptoms of trauma or PTSD or a review evaluating such studies. Exclusion criteria were the absence of evaluation of trauma symptoms or PTSD. No restrictions were set on language, type of publication, or year of publication. The search was conducted on January 28th, 2022, a second reviewer repeated it on March 11th, 2022, and a third review on January 13th, 2023. The initial searches returned 70 records from the first reviewer and 56 from the second.

### Study selection

**Figure 1:**
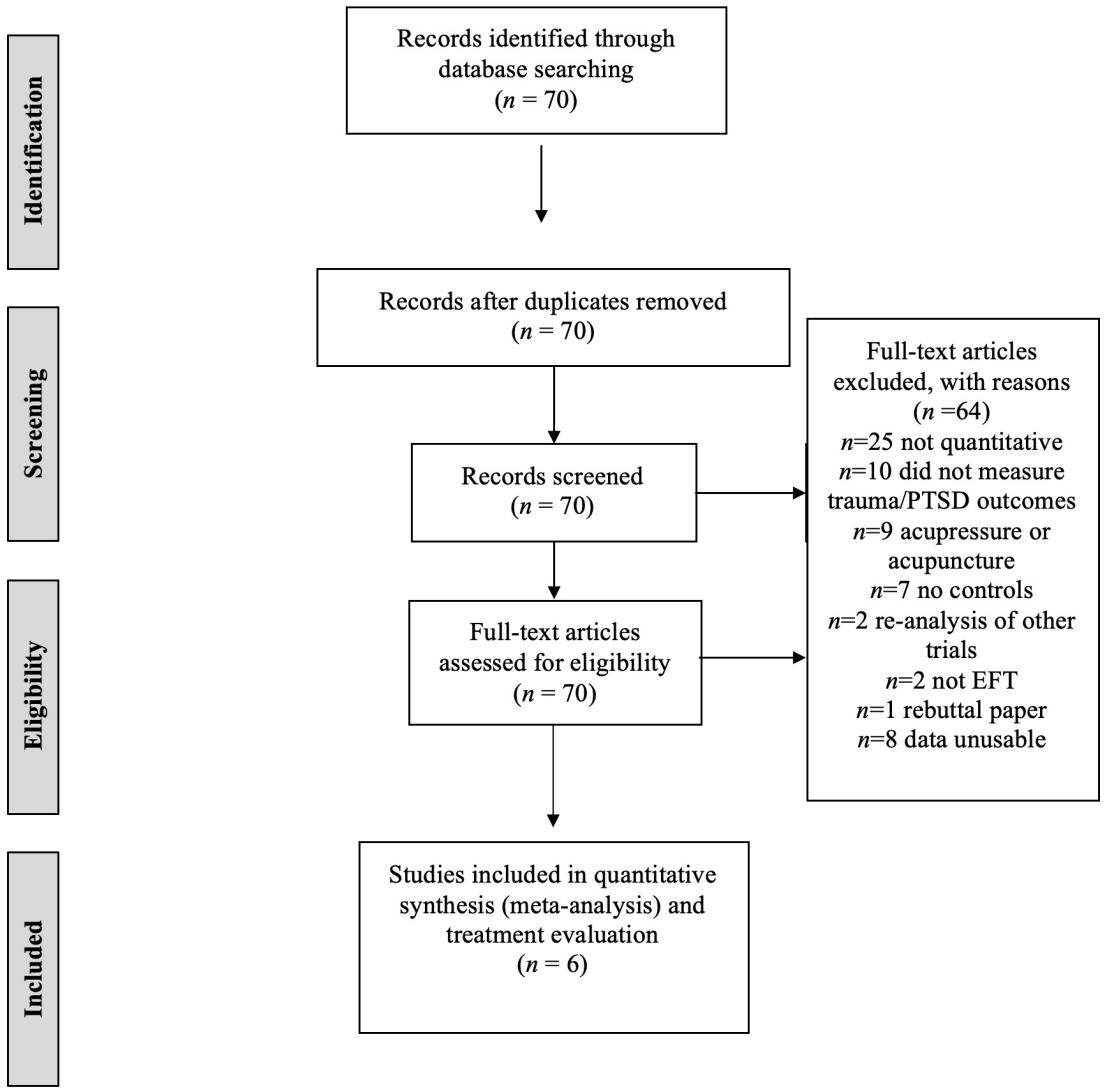
Flow chart for EFT search process. This figure illustrates the search process for locating reviews eligible for inclusion. EFT, Emotional Freedom Techniques for trauma and PTSD.

Reviewers double coded each of the 70 records as eligible, not eligible, or possibly eligible based on the title and abstract. For the systematic reviews or the single meta-analysis, full texts were obtained and searched for eligibility. All discrepancies were resolved via consensus between the two reviewers. Of the 70 records, 56 were excluded as not meeting the study criteria. Ten trials did not specifically measure trauma or PTSD symptoms; nine employed an acupressure or acupuncture intervention other than Clinical EFT; two papers were a re-analysis of other trials; seven trials did not have comparison or control groups; 25 papers were reviews only; two trials employed tapping interventions other than Clinical EFT; and one paper was a critique of a quantitative study. Further inspection of the data resulted in the identification of eight trials where, despite randomization, no data was available on the treatment as usual comparison group before allocation to the intervention. Thus, seven papers were found eligible for review (see Figure 1). For the six included papers, committee members coded the PICTOS (population, intervention, comparison, outcomes, timeline and setting) criteria. Delivery format (individual versus group EFT) was not limited, as previous research has indicated similar outcomes between the two styles (Church et al., 2022). Because less therapist time is needed for group intervention, it has the advantage of being cost effective. See Table 1 for details.

**Table 1:** Studies included in the review of clinical EFT.

Study	Intervention(s)	Population	Setting	Comparison condition	Sample	Outcome	Measure employed	Time points
1 Al-Hadethe et al., 2015	EFT	Male students 16–19 years	Baghdad city	Narrative exposure therapy (NET); control group	20 EFT; 20 NET; 20 control	PTSD symptoms	Scale of posttraumatic stress symptoms	Post treatment; 3-months follow-up; 6-months follow-up; 12-months follow-up
2 Church et al., 2013	Individual EFT	Veterans	Mental health services	Standard care waitlist	30 EFT; 29 standard care	PTSD symptoms	Posttraumatic checklist–military	Post treatment; 6-month follow-up
3 Church et al., 2016	Individual EFT	Sub clinical veterans	Private practice	Treatment as usual	12 EFT; 9 TAU	PTSD symptoms	Posttraumatic checklist–military	Post treatment; 3-month follow-up; 6-month follow-up
4 Geronilla et al., 2016	Individual EFT	Veterans	Clinical setting; or telephone or televideo conferencing	Treatment as usual	32 EFT; 26 TAU	PTSD symptoms	Posttraumatic checklist–military	Post treatment; 3-month follow-up; 6-month follow-up
5 Karatzias et al., 2011	Individual EFT	Adults diagnosed with PTSD	Clinical setting	EMDR	23 EFT; 23 EMDR	PTSD symptoms	Posttraumatic checklist–civilian	Post treatment; 3-month follow-up
6 Nemiro and Papworth, 2015	Group EFT	Female refugees who were victims of sexual gender violence, Democratic Republic of Congo	Centre for displaced women	Cognitive behavioral therapy	25 EFT; 25 CBT	PTSD Symptoms	Harvard Trauma questionnaire	Post treatment; 6-month follow-up

Due to methodological issues with several studies that were included in the earlier meta-analysis, we excluded two from this analysis. Specifically, Church et al. (2012) did not directly measure PTSD, but rather the Impact of Events scale. Church et al. (2015) was also excluded (reported as a conference proceeding in the earlier meta-analysis,) because despite randomization, there was no data available on treatment-as-usual group before crossover to EFT in the more recent published paper (Church et al., 2018b).

### Data extraction and coding

All studies were evaluated for outcome variables by two reviewers. Any discrepancies were resolved through consensus. Outcomes included PTSD symptomology, other trauma-related symptomology, and diagnosis of PTSD.

### Statistical analysis

The six qualifying controlled trials were stratified by those that compared EFT to treatment as usual (TAU) or waitlist control, and those that compared EFT to an evidence-based alternative psychotherapy. The trial by Al-Hadethe et al. (2015) had both active treatment and no treatment control conditions, and thus was separately included in both sets of studies. Thus, there were 4 trials that compared EFT to untreated controls and 3 trials that compared EFT to active treatment controls. In a separate sensitivity analysis, the Al-Hadethe et al. (2015) trial was removed from the meta-analysis that compared EFT to TAU and a separate aggregate effect sizes was calculated (i.e., *n* = 3 studies evaluated).

For each trial, the effect size of EFT versus control condition was calculated by use of Hedge’s *g* along with its 95% confidence interval. The summary (aggregate) effect size estimates across trials were calculated with both fixed and random effect methods using the Comprehensive Meta-Analysis (CMA) software program (Biostat Inc., Englewood, NJ, United States). The aggregate effect size calculated across studies in this meta-analysis is a “weighted” mean rather than simple arithmetic mean, thus the weight of each study is derived by its sample size and for the random effects, for how its result compares to results from the other studies. Presenting a simple arithmetic mean could be potentially very misleading, and again, does not take into account the fundamental differential weighting of the meta-analysis which is paramount. Assessment or heterogeneity across trial results (i.e., beyond chance) from the summary estimates were calculated by the I^2^ statistic (Higgins and Thompson, 2002).

### Risk of bias

For each of the included RCTs, two committee members coded the quality of methodology according to the Cochrane Reviews (Higgins et al., 2022), which is the recommended tool to assess the risk of bias in randomized trials. The judgment about the risk of bias arising from each domain is generated by an algorithm, based on answers to the signaling questions. Judgement was made to be “Low” or “High” risk of bias, or “Some Concerns.”

Table 2 summarizes the judgments made for the six included trials. The majority of risk of bias domains were rated low, with “some concerns” listed mainly for domain 2. In particular this was due to the query in *2.2. Were carers and people delivering the interventions aware of participants’ assigned intervention during the trial?* It is obvious that in psychotherapy trials, therapists cannot be blind to the interventions they are delivering. Nonetheless, the Cochrane criteria require that if a single rating of “some concerns” occurs, then the overall rating should be the same, despite the other domains being low.

**Table 2:** Overall risk of bias for current studies.

Study	Domain 1	Domain 2	Domain 3	Domain 4	Domain 5	Overall rating
1 Al-Hadethe et al., 2015	Low	Some	Low	Low	Low	Low/Some concerns
2 Church et al., 2013	Low	Low	Some	Low	Low	Low/Some concerns
3 Church et al., 2016	Low	Low	Some	Low	Low	Low/Some concerns
4 Geronilla et al., 2016	Low	Some	Low	Low	Low	Low/Some concerns
5 Karatzias et al., 2011	Low	Some	Low	Some	Some	Low/Some concerns
6 Nemiro and Papworth, 2015	Low	Some	Low	Low	Some	Low/Some concerns

Publication bias was not performed due to the limited range of studies available and is discussed as a limitation.

## Results

**Figure 2:**
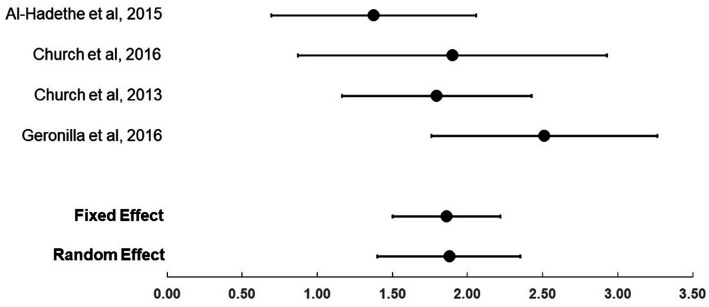
Effect sizes (Hedge’s g)and 95CIs comparing EFT to no treatment controls.

For the four trials that compared EFT (total *n* = 88) to no treatment controls (total *n* = 76), the effect sizes (Hedge’s *g*) were large, ranging from 1.38 to 2.51 (see Table 3; Figure 2). The fixed and random effect summary estimates were similar at 1.86 and 1.88, respectively, and both methods were highly statistically significant (*p* < 0.001). Heterogeneity of trial results was considered moderate, with an *I*^2^ value of 41.1. In the sensitivity analysis that removed the Al-Hadethe et al. (2015) trial, the fixed effect size was 2.06 (95% confidence interval: 1.62–2.49, *p* < 0.001).

**Table 3:** Effect Sizes of Trials Comparing EFT to TAU/Waitlist for Treatment of PTSD Symptoms.

Study	#	N*	Pre-intervention				Post-intervention				Effect (ES) size estimates			
			EFT		TAU/Waitlist		EFT		TAU/Waitlist		ES	Lower CL	Upper CL	value of *p*
			*M*	SD	*M*	SD	*M*	SD	*M*	SD				
Al-Hadethe et al., 2015	1	20/20	23.6	8.05	22.95	8.95	13.65	5.38	25.05	10.19	1.38	0.69	2.06	<0.001
Church et al., 2016	2	12/9	41.0	8.0	36.0	6.0	25.0	8.3	36.0	9.0	1.90	0.87	2.93	<0.001
Church et al., 2013	3	29/25	62.01	11.31	62.71	11.5	39.41	14.54	63.23	10.0	1.80	1.17	2.43	<0.001
Geronilla et al., 2016	4	27/22	65.0	8.1	67.0	7.8	34.0	10.3	63.0	10.4	2.51	1.76	3.26	<0.001

**Fixed Effect**	All	88/76	-----	1.86	1.50	2.22	<0.001
**Random Effect**	All	88/76	-----	1.88	1.40	2.35	<0.001

*EFT/Comparison group sample sizes used in the analysis. EFT, emotional freedom technique; TAU, treatment as usual; M, mean; SD, standard deviation; CL, confidence limit. The heterogeneity I^2^ for the 4 trials was 41.1.

**Figure 3:**
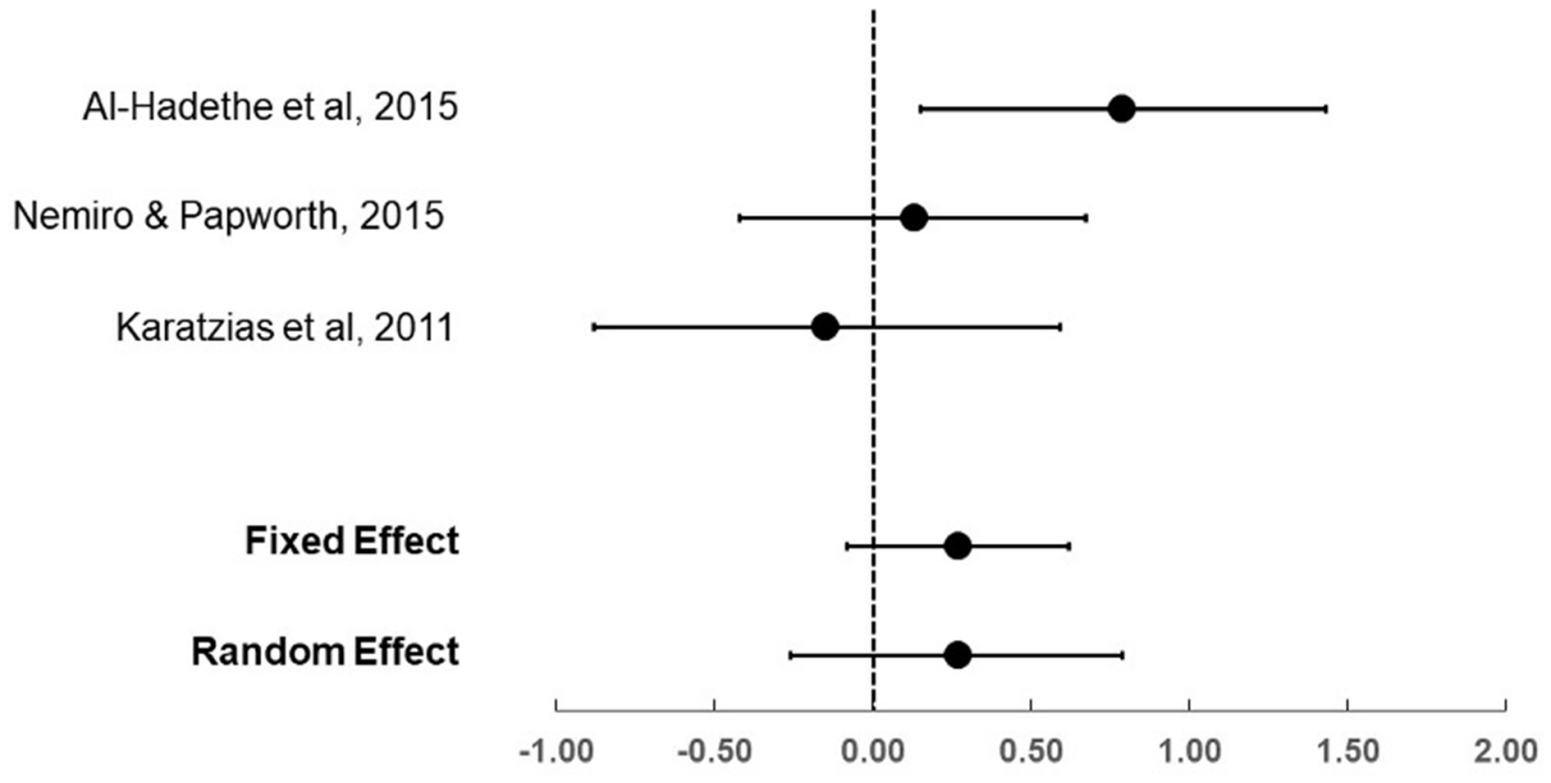
Effect sizes (Hedge’s g)and 95%CIs comparing EFT to active treatment controls.

For the three trials that compared EFT (total *n* = 58) to active treatment controls (total *n* = 58), the effect sizes (Hedge’s *g*) ranged from −0.15 to 0.79 (see Table 4; Figure 3). The fixed and random effects summary estimates were both 0.27, and neither estimate was statistically significant, suggesting similar results between Clinical EFT and the comparator evidence-based therapies (EMDR, NET and CBT). Heterogeneity of trial results was considered moderate, with an *I*^2^ value of 54.1.

**Table 4:** Effect sizes of trials comparing EFT to other evidence-based psychotherapies for treatment of PTSD symptoms.

Study	#	N*	Pre-intervention				Post-intervention				Effect (ES) size estimates			
			EFT		Other Tx**		EFT		Other Tx**		ES	Lower CL	Upper CL	value of *p*
			*M*	SD	*M*	SD	*M*	SD	*M*	SD				
Al-Hadethe et al., 2015	1	20/19	23.6	8.05	23.31	8.52	13.65	5.38	18.26	6.56	0.79	0.15	1.43	0.02
Nemiro and Papworth, 2015	5	25/25	2.54	0.42	2.71	0.57	1.59	0.41	1.83	0.60	0.13	−0.42	0.67	0.65
Karatzias et al., 2011	6	13/14	57.8	12.0	59.3	11.0	42.0	16.9	41.6	21.8	−0.15	−0.88	1.59	0.70

**Fixed Effect**	All	58/58	-----	0.27	−0.08	0.62	0.13
**Random Effect**	All	58/58	-----	0.27	−0.26	0.79	0.32

*EFT/Comparison group sample sizes used in the analysis. **The Other Treatments (Tx) were Narrative Exposure Therapy (NET), Cognitive Behavior Therapy (CBT), and Eye Movement Desensitization Reprocessing (EMDR) for studies 1, 5, and 6, respectively. EFT, emotional freedom technique; M, Mean; SD, standard deviation; CL, confidence limit. The heterogeneity I^2^ value for the 3 trials was 54.1.

## Discussion

Therapies that integrate a somatic component into treatment, such as EMDR and EFT, have faced obstacles to widespread adoption in clinical settings. While they have been validated in over 100 clinical trials each, their evidence base is smaller than that of talk therapies such as cognitive therapy which have been practiced for decades longer. Nonetheless, a growing body of research suggests that their effects for PTSD, anxiety, and depression are robust, with participant gains maintained on long-term follow-up. The current updated review demonstrates that Clinical EFT produces greater reduction in PTSD symptoms than wait-list or “treatment-as-usual” control groups, symptom reductions similar to other evidence-based therapies, and large treatment effects. The physiological mechanisms of action of Clinical EFT have been elucidated in the dimensions of stress hormone reduction, gene expression, brain regulation, and biomarkers such as heart rate and blood pressure.

Clinical EFT is classified as a “generally safe therapy” by the US Veterans Administration (Church et al., 2017). Additional evidence for its safety is provided by the adverse events reporting from clinical trials, which have identified no adverse events in studies involving more than 2,000 participants (Church et al., 2022). Conversely, the amelioration of emotional distress in brief time frames is noted (Flint et al., 2005) and rapid treatment effects are one of the characteristics of Clinical EFT for PTSD (Feinstein, 2022a). An exploration of therapists’ experiences using such methods for adult survivors of childhood sexual abuse (Schulz, 2007) reports that clients prefer the intervention because it lessens the possibility of re-traumatization.

### Limitations

There are several limitations to this review. The number of studies was modest and sample sizes were small, which may have larger effects than larger studies would, which can lead to overestimations of the true effect size. Publication bias may have also occurred, although it is unknown how many unpublished studies may exist. Future reviews may benefit from graphical representations of the relationship between effect size and study precision (usually represented by the standard error or sample size in a funnel plot), or Egger’s regression test to formally test for publication bias in addition statistically robust estimates of the true effect of the clinical EFT intervention, underpinned by stronger datasets. It is acknowledged publication bias is an inherent limitation of any meta-analysis.

The trials included in this meta-analysis all employed self-report measures to evaluate the presence of PTSD and reduction of symptoms. While the majority utilized the PTSD Checklist (PCL), the most common instrument for assessing symptom change, screening individuals for PTSD and making a provisional PTSD diagnosis, an observer-rated measure such as the Clinician-Administered PTSD Scale (CAPS-5) is strongly recommended for future research.

Not every trial stated that the intervention was carried out with fidelity to the treatment modality, though all did use the manualized version of the method (Church, 2013b). A further limitation derives from the origin of the Cochrane standards in trials of pharmaceutical drugs. In our ROB analysis, the majority of risk of bias domains were rated low. However, for domain 2, the rating was “some concerns.” This was due to the wording of question *2.2. Were carers and people delivering the interventions aware of participants’ assigned intervention during the trial?* In psychotherapy trials, the therapists “delivering the interventions” cannot be blind to the assigned intervention.

Therapists have to be trained in the method being studied in order to treat clients. Ethical standards typically require training and expertise in a therapy prior to using it to treat others. Therefore, it is impossible to conduct a blind psychotherapy trial in which the therapist is unaware of which method he or she is using. While this standard is useful in pharmacology trials, it leads to skewed results in psychotherapy trials. The Cochrane criteria state that if a single rating of “some concerns” occurs for a domain, such as is inevitable for item 2.2, then the overall rating for that domain should be the same. Had the Cochrane rating been adjusted for this factor, all studies would have considered as low risk of bias.

Mitigating the above limitations, this review has several strengths. The quantitative meta-analysis was conducted by an independent research academician (KK) without any allegiance to the intervention. The secondary searches (TO) were conducted by a research clinician also independent of allegiance to the method; ROB evaluations were completed by academics (DC and LT), one of whom is likewise independent (LT). Two further authors (JF and PB) represent a training organization for the intervention which consulted on the NICE evaluation. The evaluation was led by an academic researcher (PS) with experience and training in systematic reviews and although both PS and DC do conduct clinical trial research in the modality under examination, our goal was to conduct the review transparently. PS does not conduct trials in the area of PTSD and no studies were included with their authorship, thus was considered the most appropriate academic to lead the report. Overall, our goal was to conduct the review transparently. While we recommend an update when further studies are available, extant research indicates a large treatment effect for Clinical EFT for PTSD.

## Conclusion

Numerous randomized controlled trials and outcome studies, as well as a meta-analysis, have demonstrated Clinical EFT to be an effective evidence-based treatment for PTSD. The APA Division 12 Task Force for Empirically Validated Therapies published a set of standards by which to evaluate therapies in the late 1990s. Earlier reviews (Feinstein, 2012; Church, 2013a; Church et al., 2022) found that Clinical EFT meets these standards.

## Data availability statement

The original contributions presented in the study are included in the article/supplementary material, further inquiries can be directed to the corresponding author.

## Author contributions

PS and TO’K conducted the searches and all studies were evaluated for outcome variables. PS and DC oversaw the design, analysis, and writing of the paper. KK completed the meta analysis. LT and DC completed the risk of bias analysis. All authors contributed to the article and approved the submitted version.

## Funding

The present study was partially supported by an internal Faculty grant from Bond University to the author PS.

## Conflict of interest

PS declares she may lead clinical research trials in the topic. DC declares that he receives remuneration from publications and presentations on the therapeutic approach examined.

The remaining authors declare that the research was conducted in the absence of any commercial or financial relationships that could be construed as a potential conflict of interest.

## Publisher’s note

All claims expressed in this article are solely those of the authors and do not necessarily represent those of their affiliated organizations, or those of the publisher, the editors and the reviewers. Any product that may be evaluated in this article, or claim that may be made by its manufacturer, is not guaranteed or endorsed by the publisher.

## Footnotes

^1^https://www.nice.org.uk/guidance/ng116/evidence, “Evidence Review D,” retrieved October 13, 2022.
